# Efficiency of Barley Bran and Oat Bran in Ameliorating Blood Lipid Profile and the Adverse Histological Changes in Hypercholesterolemic Male Rats

**DOI:** 10.1155/2013/263594

**Published:** 2013-08-01

**Authors:** Haddad A. El Rabey, Madeha N. Al-Seeni, Hanan M. Amer

**Affiliations:** ^1^Biochemistry Department, Faculty of Science, King Abdulaziz University, Jeddah, Saudi Arabia; ^2^Bioinformatics Department, Genetic Engineering and Biotechnology Research Institute, Minufiya University, P.O. Box 79, Sadat City, Egypt; ^3^Badreddin Clinic, Jeddah, Saudi Arabia

## Abstract

The efficiency of oat bran and barley bran in lowering the induced hyperlipidemia and hypercholesterolemia in blood of male Albino rats (*Rattus rattus*) was studied. Twenty rats were divided into four groups each consisted of five rats and fed the specified test diets for eight weeks. The first group (G1) is the negative group which was fed basal diet, the second group (G2) was fed 1.0% cholesterol, was the third group (G3) fed 1.0% cholesterol and 10% oats bran, and the fourth group (G4) was fed 1.0% cholesterol and 10% barley bran. Feeding rats on 1% cholesterol significantly increased serum total cholesterol, low density lipoprotein, and very low density lipoprotein and triglyceride and decreased serum high density lipoprotein. Furthermore, enzyme activity of alanine aminotransferase, aspartate aminotransferase, and alkaline phosphatase was increased, and lipid peroxide was increased, whereas catalase and glutathione-S-transferase were decreased. Kidney functions parameters in the cholesterol supplemented group were elevated compared with the negative control. In addition, histological alteration in kidney, liver, heart, and testes was observed, compared with the negative control. Hypercholesterolemic rats supplemented with oat bran and barley bran showed significant decrease in lipid parameters, significant increase in high density lipoprotein-cholesterol, improved antioxidant enzyme, and improved histopathology of kidney, liver, heart, and testes. In conclusion, both oat bran and barley bran had protective effects against induced hyperlipidemia and improved histological alterations. Oat bran appeared more efficient than barley bran in lowering the lipid profile levels in hypercholesterolemic rats.

## 1. Introduction

Dietary or pharmacological reduction of total and low density lipoproteins (LDL) decreases the risk of coronary heart diseases (CHD) [1]. LDL is the major atherogenic lipoprotein, and numerous clinical trials have shown the efficacy of lowering low density lipoproteins-cholesterol (LDL-C) for reducing CHD risk [2]. Consumption of diets rich in whole grains has been reported to have beneficial health effects such as reducing the risk of cancer, cardiovascular disease, and noninsulin-dependent diabetes mellitus. These results have been attributed to the effects of the fiber content of whole-grain foods on risk factors of these diseases, including blood glucose, insulin, and cholesterol [3]. Numerous studies have demonstrated that whole grains that are high in soluble fiber, such as oats and barley, are more effective in lowering blood cholesterol than grains in which fibers are predominantly insoluble, such as wheat or rice [4, 5].

Increasing dietary fiber, which is a variety of plant substances that are resistant to digestion by human gastrointestinal enzymes, has been used for cholesterol reduction [6, 7]. Moreover, dietary fibers can be classified into two major groups depending on their solubility in water in human body, and the structural or matrix fibers (lignins, cellulose, and some hemicelluloses) are insoluble, whereas the natural gel-forming fibers (pectins, gums, mucilages, and the remainder of the hemicelluloses) are soluble. Studies have focused on soluble fibers such as oats, psyllium, pectin, barley, and guar gum, and qualitative reviews suggested that these fibers lower total and LDL-C [6, 7].

*β*-glucan is a nonstarch polysaccharide composed of beta-(1-4)-linked glucose units separated every 2-3 units by beta-(1-3)-linked glucose [8]. This soluble fiber, which is found in oat and barley, has a potential to reduce LDL-C through the increase in intestinal viscosity that may lower cholesterol absorption, although reduced cholesterol absorption was not found in other studies [8, 9].

Oat is an important source for water-soluble fibers, and the beneficial effects of oat products on the lipoprotein profile are ascribed to their soluble fiber compound, *β*-glucan [10]. In addition, oat is a source of antioxidants, such as tocols and various phenolic compounds [11]. Oat antioxidants have been reported to inhibit low-density lipoprotein oxidation and promote scavenging of reactive oxygen species [12, 13]. There are many studies indicating the efficacy of oat bran in reducing total cholesterol (TC) and LDL-C concentrations while either increasing or having no effect on plasma HDL-C concentrations in humans [10, 14, 15].

Barley, like oat, is a rich source of the soluble fiber *β*-glucan, which has been shown to significantly lower LDL-C [16]. The effects of barley *β*-glucan on cardiovascular and diabetic risk were reviewed [17]. Concentrated *β*-glucan extracts with high or low molecular weight added to food products were reported efficiently to lower LDL-C in hyperlipidaemic subjects with and without the metabolic syndrome [18]. The consumption of barley and its products might reduce many risk factors associated with the metabolic syndrome, diabetes, hypertension, and dislipidemia [19].

The purpose of this study is testing the efficiency of barley bran and oat bran (as *β*-glucan containing nutrients) in lowering blood cholesterol levels and ameliorating the histological changes (resulted from hypercholesterolemia) of kidney, liver, heart, and testes in male rats.

## 2. Materials and Methods

### 2.1. Animals and Housing Conditions

Twenty male Albino Wister rats “*Rattus rattus*” weighing about 140–160 g were obtained from King Fahd Medical Research Center, King Abdulaziz University, Jeddah, Saudi Arabia. Rats were housed five per polycarbonate cages. Cages, bedding, and glass water bottles (equipped with stainless steel sipper tubes) were replaced twice per week. Stainless steel feed containers were changed once per week. Oats and barley were obtained from a Grain Silos and Flour Mills. The seeds were milled and then sieved to get the bran. The animals were kept at room temperature (25 ± 5°C) with a natural lighting cycle (12 hours), fed a standard basal diet [20], and kept under observation for 2 weeks before the start of the experiment to exclude any undercurrent infection.

### 2.2. Experiment Design

The animals were then divided randomly into four groups each of 5 rats as follows: group 1 (G1): fed normal diet, group 2 (G2): fed 1.0% w/w cholesterol ex-pure, from Oxford Laboratory Reagent Company (Egypt) in diet to induce hypercholesterolemia [21], group 3 (G3): fed cholesterol as in (G2) and supplemented with 10% oat bran [22], and group 4 (G4): fed cholesterol as in (G2) and supplemented with 10% barley bran to be equal to that of oat bran.

The trial period of the current study was 8 weeks because it has been proven to be the most efficient period in causing hypercholesterolemia [23, 24]. At the 8th week blood samples were collected from all rats after anaesthetizing them by dimethyl-ether, and then blood samples were collected from the ophthalmic orbital sinus [25]. It is generally accepted that the most reliable data on blood lipid metabolism can be obtained from fasting animals, 14–16 hours after their last feeding. Therefore, food was removed from the cages at 6 p.m. a day before blood samples were drawn, and the samples were collected at 9 a.m. the next day.

### 2.3. Biochemical Tests

Blood was collected in EDTA tube for CBC analysis and in plain tubes for chemistry analyses. Serum was obtained by blood samples centrifugation at 1000 rpm for 10 min at room temperature and then stored at −20°C until analysis was performed. All biochemical analyses (lipid profile tests “sTC, sTG, sLDL-C, sHDL-C, and sVLDL-C,” liver enzymes “AST, ALT, and ALP,” and kidney function parameters “creatinine, urea, and uric acid”) were achieved using the specified kits from Gesellschaft for Biochemical and Diagnostic (Germany) according to the instructions of the suppliers. At the end of the experiment and after collection of blood, anaesthetized animals were scarified by cervical dislocation. The abdomen was opened, and the organs were rapidly excised. The left kidney was quickly rinsed in isotonic saline and dried on a piece of filter paper. One hundred gram of kidney tissue was homogenized in ice-cold saline using a glass homogenizer. The homogenate was then diluted with the homogenization medium to ultimately yield 10% (v/v). Biodiagnostic Chemical Company (Egypt) kits were used in estimating GST [26], catalase [27], and lipid peroxide [28] in the kidney homogenate according to the instructions of the suppliers.

### 2.4. Histopathological Investigations

Liver, heart, right kidney, and testis were washed in sterile saline and fixed in 10% neutral formalin for histopathological studies. The target organs were then dehydrated in gradual ethanol (50–99%), cleared in xylene, and embedded in paraffin. Sections were prepared and then stained with hematoxylin and eosin (H&S) dye for microscopic investigation [29]. The stained sections were examined and photographed under a light microscope.

### 2.5. Statistical Analysis

All data were analyzed using the SPSS (Statistical Program for Sociology Scientists) Statistics Version 17.0 for computing the mean values, the standard errors (SE), and test of significance (*t*-test).

## 3. Results

### 3.1. The Effect of Tested Diets on Lipid Profile

The effect of oat bran and barley bran supplementation, for 8 weeks on serum lipids in rats with induced hypercholesterolemia is illustrated in Table 1. As shown, the mean values of s.TC, s.TG, s.LDL-C, and serum very low density lipoprotein-cholesterol (s.VLDL-C) in the positive control were higher than that of the negative control, whereas the serum HDL-C was decreased. The differences were highly significant (*P* < 0.001), in s.TC, S.TG, s.LDL-C, and HDL-C compared with that of the negative control. In G3 and G4, the mean values of the s.TC, s.TG, S.LDL-C, and s.VLDL-C were lower than that of the positive control. The differences were highly significant (*P* < 0.001) compared with the positive control.

### 3.2. The Effect of Tested Diet on Liver Enzymes

Table 2 shows the effect of oat bran and barley bran supplementation for 8 weeks on liver enzymes in rats with induced hypercholesterolemia. The mean values of ALT, AST, and ALP in the positive control were higher than that of the negative control. The differences were significant (*P* < 0.05) in case of ALT, nonsignificant in case of AST, and highly significant (*P* < 0.001) in case of ALP. In G3 and G4, the mean values of ALT, ALP, and AST were lower than that of the positive control.

### 3.3. Effect of the Tested Diets on Kidney Function

The effect of barley bran and oat bran supplementation for 8 weeks on kidney function in rats with induced hypercholesterolemia is given in Table 3. The mean values of serum uric acid and urea in the positive control were higher than that of the negative control. The differences were significant (*P* < 0.05) in case of uric acid and highly significant (*P* < 0.01) in case of urea, compared with the negative control. While the values of uric acid and urea in G3 and G4 were lower than that of the positive control, differences were nonsignificant in case of uric acid while being highly significant (*P* < 0.01) in case of urea compared with the positive control. The mean values of creatinine in the positive control were nonsignificantly higher than that of the negative control, while in G3 and G4, the mean values were lower than that of the positive control.

### 3.4. Effect of the Tested Diets on GST, Catalase, and Lipid Peroxide

The effect of oat bran and barley bran supplementation for 8 weeks on catalase, GST, and lipid peroxide in kidney homogenate of rats with induced hypercholesterolemia is shown in Table 4. The mean values of GST in the positive control were lower than that of the negative control. While the mean values of catalase and lipid peroxide were higher than that of the negative control, the differences were highly significant (*P* < 0.001) in case of GST and catalase compared with the negative control. In G3 and G4, the mean values of GST and catalase were higher than that of the positive control. While the mean values of lipid peroxide were lower than that of the positive control, the differences of catalase mean values in G3 and G4 were highly significant (*P* < 0.001), whereas differences in mean values of lipid peroxide in G4 were significant (*P* < 0.05) compared with the positive control.

### 3.5. Histopathological Investigation

#### 3.5.1. Kidney

**Figure 1:**
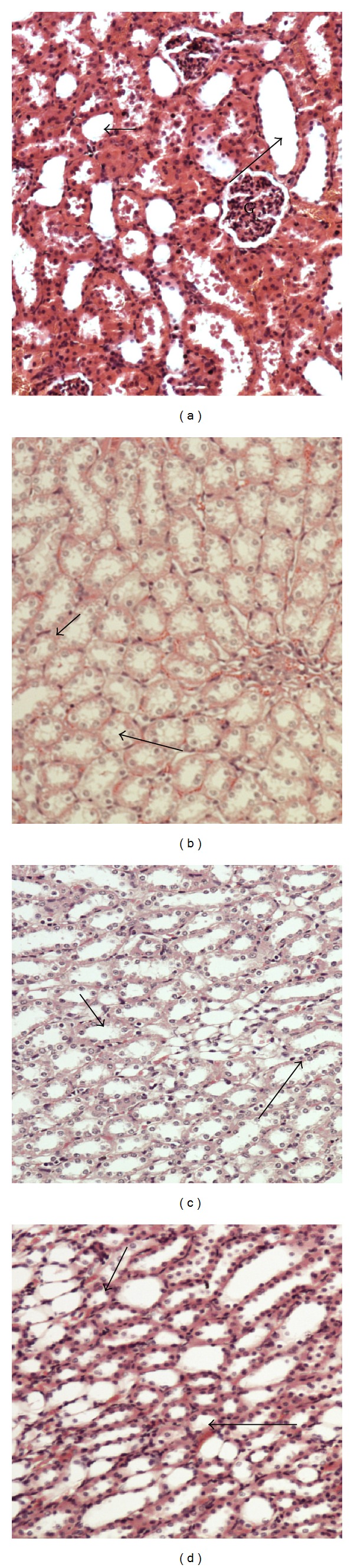
Effect of barley bran and oat bran supplementation for 2 months on renal tissue structure in rats with induced hypercholesterolemia. (a) Kidney renal tissues of control group showing normal renal structure with regulated nuclear arrangement of uriniferous tubules (small arrow) and collecting tubules (long arrow) with glomerulus (G), (b) renal tissues of hypercholesterolemic group showing bale renal tissues showing disrupted small uriniferous tubule (short arrow) and small Bowman's capsule with dilated urinary space. The renal tubules appeared irregular, dilated, and shrinkage of the glomerulus which led to dilated urinary and collecting tubule space (long arrow), (c) renal tissues of oat bran-treated group showing convoluted urineferous tubules (small arrow) with certain arranged collecting tubules (long arrow), and (d) renal tissues of barley bran supplementation: the sections looked to be restoring the normal appearance, and the deposition of cholesterol was decreased in the kidney which showed well-defined urinary tubules (short arrow) and collecting tubules (long arrow). ×200 (H&E stains).

Normal renal tissues of control group is shown in Figure 1(a). Normal renal structure with regulated nuclear arrangement of uriniferous tubules and collecting tubules with glomerulus (G) is observed.  Figure 1(b) shows renal tissues of hypercholesterolemic group with bale renal tissues showing disrupted small uriniferous tubule and small Bowman's capsule with dilated urinary space. The renal tubules appeared irregular, dilated, and shrinkage of the glomerulus which led to a dilated urinary and collecting tubule space. Figure 1(c) shows renal tissues of oat bran-fed-group with convoluted uriniferous tubules with certain arranged collecting tubules.  Figure 1(d) shows renal tissues of barley bran supplementation, the sections looked to be restoring the normal appearance, and the deposition of cholesterol was decreased in the kidney which showed well-defined urinary tubules and collecting tubules.

#### 3.5.2. Liver

**Figure 2:**
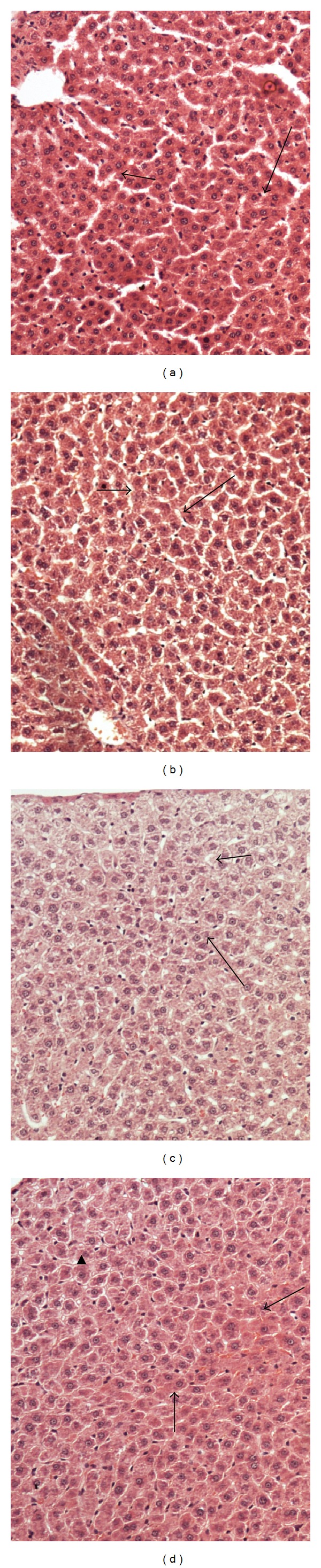
Effect of barley bran and oat bran supplementation for 2 months on liver tissue structure in rats with induced hypercholesterolemia. (a) Hepatic tissues of control group showing hepatic strands of cells (short arrow) blood sinusoids (long arrow), (b) hepatic tissue suffering hypercholesterolemia showing fatty liver tissue with disrupted cells, vacuolated cytoplasm, and necrosis with disrupted hepatic strands (arrows), (c) hepatic tissues pretreated oat bran shows be restoring the normal appearance of hepatic strands (short arrow), well-defined hepatic cords with polyhedral hepatocytes, and normal appearing round nuclei (long arrow), and (d) hepatic tissues pretreated barley bran shows no necrotic hepatic tissues with normal hepatic strands (arrows). ×200 (H&E stains).

Hepatic tissues of control group with hepatic strands of cells and blood sinusoids are given in Figure 2(a).  Figure 2(b) shows that the hepatic tissue suffering from hypercholesterolemia has fatty liver tissue with disrupted cells, vacuolated cytoplasm, and necrosis with disrupted hepatic strands.  Figure 2(c) shows that the hepatic tissues of rats-fed oat bran restored the normal appearance of hepatic strands. Moreover, well-defined hepatic cords with polyhedral hepatocytes and normal appearing round nuclei are also shown.  Figure 2(d) shows hepatic tissues of rats fed barley bran with no necrotic hepatic tissues with normal hepatic strands.

#### 3.5.3. Testes

**Figure 3:**
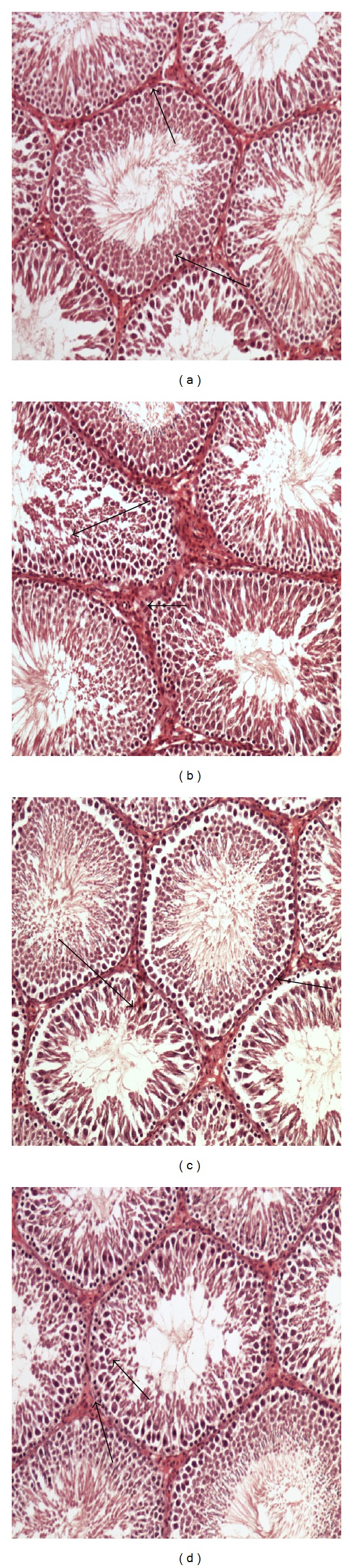
Effect of barley bran and oat bran supplementation for 2 months on testicular structure in rats with induced hypercholesterolemia. (a) Control rats showing normal and regular seminiferous tubules (arrows), (b) hypercholesterolemia group showing the pathologic effects in which the structures of the seminiferous tubules were severely damaged, and thickness of the tubular walls was increased (short arrow) and number of germinal cells was greatly decreased with a disturbance in their diameter (long arrow), (c) oat bran-treated group showing normal and regular seminiferous tubules, and germinal cells were greatly regulated with normal diameter and mild dilatation of the seminiferous tubules with normal complete spermatogenic series (short arrow) with normal thickness (long arrow), and (d) barley bran-treated group showing normal seminiferous tubules with normal germinal cell layers (arrows). ×400 (H&E Stain).

The testicular structure of the testes of control rats with normal and regular seminiferous tubules is shown in Figure 3(a).  Figure 3(b) shows the hypercholesterolemic group suffering pathologic effects in which the structures of the seminiferous tubules were severely damaged and the thickness of the tubular walls was increased. The number of germinal cells was greatly decreased with a disturbance in their diameter.  Figure 3(c) shows oat bran-fed group with normal and regular seminiferous tubules. The germinal cells were greatly regulated with normal diameter and mild dilatation of the seminiferous tubules with normal complete spermatogenic series with normal thickness.  Figure 3(d) shows barley bran-fed group which has normal seminiferous tubules with normal germinal cell layers (arrows).

#### 3.5.4. Heart

**Figure 4:**
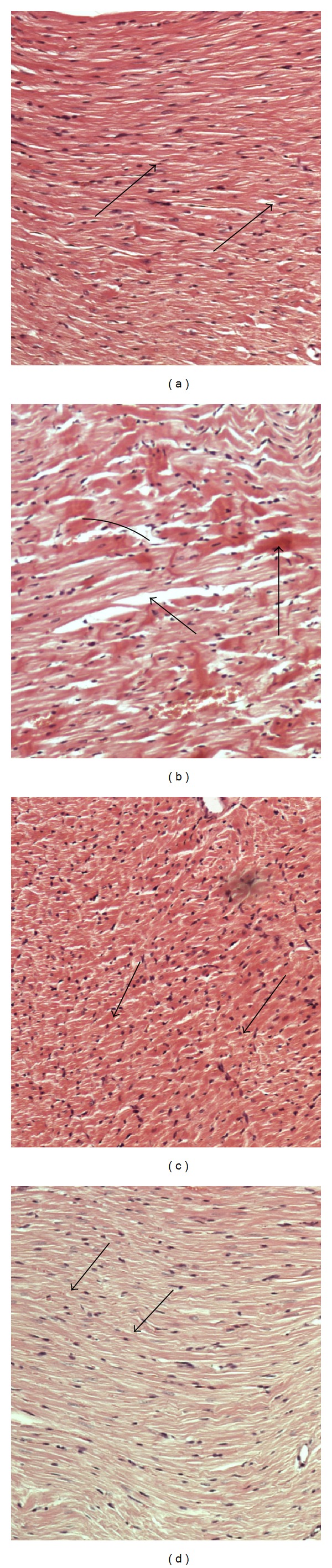
(a) Cardiac tissues of control group showing normal structure of cardiac muscles consist of muscle fibers (arrows), (b) cardiac tissues of hypercholesterolemia group showing increased hyalinization (short arrow) with cardiac muscles damage (long arrow) and necrosis of muscle fibers (curved arrow), (c) cardiac tissues pretreated with oat bran shows minimal cardiac muscles damage with most normal tissues (arrows), and (d) pretreated with barley bran showing disappearance of pathological changes with normal cardiac tissue (arrows). ×200 (H&E stains).

Cardiac tissues of control group showing normal structure of cardiac muscles consists of muscle fibers (arrows) are shown in Figure 4(a).  Figure 4(b) shows cardiac tissues of hypercholesterolemia group showing increased hyalinization with cardiac muscles damage and necrosis of muscle fibers.  Figure 4(c) shows cardiac tissues of rats fed oat bran showing minimal cardiac muscles damage with most normal tissues.  Figure 4(d) shows cardiac tissues of barley bran-fed group showing disappearance of pathological changes with normal cardiac tissue.

## 4. Discussion

The present study has focused on testing and comparing the efficiency of two *β*-glucan containing nutrients, that is, oat bran and barley bran in ameliorating blood lipid profile. Large, prospective, epidemiologic studies proved a protective effect of dietary fiber against coronary heart disease through direct or indirect effects on serum lipids [30–32]. The reduction of cholesterol is possibly a sum of several effects; the most accepted one is due to decreased absorption of bile acids that causes a removal of steroids from the body by fecal excretion resulting in increased catabolism of cholesterol, an increase in the secretion of bile acids, a decrease in lipoprotein cholesterol secretion, and a reduction in the total body pool of cholesterol [33].

The current results showed that feeding hypercholesterolemic rats (supplemented 1% cholesterol in the diet) on 10% of barley bran or oat bran for eight weeks hindered significantly the rise of plasma lipids. This result is consistent with other investigations on animals and human [30–32], in terms of effect on total cholesterol, LDL-C, and triglyceride, whereas does not agree with the nonaffected HDL-C [30, 34]. On the other hand, the current study showed that serum HDL was significantly increased with oat and barley bran. This result is consistent with that of Anderson [35] and disagrees with that of Braaten [36], who stated that oats *β*-glucan reduced the total and LDL-C level of hypercholesterolemic adults without changing HDL-C. Aly [11] reported that the supplementation of diet with either oat or wheat bran resulted in a significant decrease in the level of serum total lipid, total cholesterol, triglycerides, LDL-C, VLDL-C, and LDL-C/HDL-C ratio with increase in the level of HDL-C compared with those fed high cholesterol. These results are concordant with our results. It is worthy to mention that the current biochemical tests results revealed that oats had better effect in reducing the level of lipids than barley.

On the other hand the present results showed that, the increase in cholesterol in cholesterol-enriched diet resulted in a significant increase in total lipids, total cholesterol, triglycerides, LDL-C, VLDL-C, and LDL/HDL ratio accompanied with decrease in HDL-C, thus providing a model for dietary hyperlipidemia. These results are in concordance with other results, on animals with nutritionally induced hypercholesterolemia [37].

The current results showed a significant increase in the level of enzyme activity of ALT, AST, and ALP in rats' serum after induction of hypercholesterolemia, compared with those received basal diet. This result is consistent with Mahfouz and Kummerow [38].

The oral administration of 1% cholesterol in the diet to rats under study increased lipid peroxide (which is an oxidative stress biomarker) and decreased GST and catalase (which are antioxidant enzymes) in the kidney homogenate, compared with the negative control. The decrease in GST and catalase levels in the kidney homogenate of cholesterol-fed rats could have a protective role against oxidation, thus preventing the formation of lipid peroxidation. In contrast, feeding hypercholesterolemic rats (fed 1% cholesterol in the diet) on diets containing 10% oat bran or barley bran showed a significant decrease in the level of lipid peroxide and increase in the level of antioxidant GST and catalase compared with those received only 1% cholesterol (G2). These results are consistent with other results suggesting protective role of dietary fiber [11, 38, 39]. 

The current investigation showed a significant increase in the level of creatinine, uric acid, and urea compared with that of the negative control. This result is consistent with other studies demonstrated a relationship between kidney disease and increased cholesterol in the diet [40–42]. In contrast, the rats fed oat bran and barley bran showed significant decrease in the level of uric acid, urea, and creatinine compared with the positive control. This result agrees with the assumption that dietary fiber improves the level of kidney function [43].

The histopathological investigations showed histological alteration in the target organs (kidney, liver, testes, and heart) in the 1% cholesterol-fed group (G2). This result supported with previous studies suggesting a correlation between hypercholesterolemia and histological changes in the organs [44–46]. On the other hand, improvement in microscopic examination of tissues in groups fed on either oat bran or barley bran together with cholesterol. Both oats bran and barley bran have a protective role against these histological alterations due to their higher content of antioxidant substance mainly beta-glucan which has powerful antioxidant attributes, as its molecules help to prevent cell damage, working in association with enzymes and reduces the effect of dietary cholesterol resulting in the increase in the GSH product by the organs [47].

It could be concluded that both oat bran and barley bran succeeded in lowering the lipid profile levels in the blood of hypercholesterolemic rats. In addition, oat bran appeared more efficient than barley bran as revealed by the different biochemical and histological investigations. The findings of our study support the addition of the barley and oat bran to human meal in order to ameliorate the blood lipid profile.

## Figures and Tables

**Table 1:** Effect of oat bran and barley bran supplementation for 8 weeks on serum lipids in rats with induced hypercholesterolemia.

Parameters	Statistics	G1	G2	G3	G4
s.TC (mg %)	Mean ± SE	92.70 ± 0.95	119.46 ± 1.10	67.80 ± 1.98	73.64 ± 3.93
	*t*-test		−13.46***	20.02***	15.15***
s.T.G (mg/dL)	Mean ± SE	93.34 ± 5.7	114.26 ± 1.67	55.52 ± 2.41	62.06 ± 6.44
	*t*-test		−2.92**	16.87***	7.79***
s.HDLc (mg/dL)	Mean ± SE	13.40 ± 0.86	8.34 ± 0.56	18.22 ± 1.24	18.98 ± 0.94
	*t*-test		5.94***	−9.12***	−10.92***
s.VLDLc (mg/dL)	Mean ± SE	17.45 ± 1.37	20.17 ± 1.67	11.10 ± 0.48	12.40 ± 1.28
	*t*-test		−1.58 NS	5.53***	2.94**
s.LDLc (mg/dL)	Mean ± SE	58.93 ± 1.54	88.12 ± 3.35	38.48 ± 2.87	42.26 ± 4.28
	*t*-test		−6.91***	10.59***	8.55***

Significant differences with controls calculated by paired sample *t*-test. *means significant at *P* < 0.05, **means highly significant at *P* < 0.01, ***means very high significant at *P* < 0.001, and NS means nonsignificant.

**Table 2:** Effect of oat bran and barley bran supplementation for 8 weeks on liver enzymes in rats with induced hypercholesterolemia.

Parameters	Statistics	G1	G2	G3	G4
ALT (U/L)	Mean ± SE	32.84 ± 2.16	38.46 ± 2.51	28.56 ± 3.15	37.34 ± 5.18
	*t*-test		−2.34*	3.62*	−2.66*
AST (U/L)	Mean ± SE	206.20 ± 19.1	263.02 ± 26.60	174.80 ± 6.74	206.80 ± 16.79
	*t*-test		1.19 NS	−0.44 NS	−1.26 NS
ALP (U/L)	Mean ± SE	182.60 ± 25.23	294.20 ± 37.66	189.20 ± 35.13	184.20 ± 20.60
	*t*-test		4.88***	−0.07 NS	1.09 NS

Significant differences with controls calculated by paired sample *t*-test. *means significant at *P* < 0.05, **means highly significant at *P* < 0.01, ***means very high significant at *P* < 0.001, and NS means nonsignificant.

**Table 3:** Effect of oat bran and barley bran supplementation for 8 weeks on kidney function in rats with induced hypercholesterolemia.

Parameters	Statistics	G1	G2	G3	G4
Uric acid mg/dL	Mean ± SE	1.96 ± 0.15	2.48 ± 0.22	1.94 ± 0.13	2.32 ± 0.16
	*t*-test		−2.58*	2.12 NS	1.24 NS
Creatinine umol/L	Mean ± SE	0.36 ± 0.06	0.38 ± 0.05	0.34 ± 0.05	0.34 ± 0.05
	*t*-test		−0.34 NS	−0.25 NS	0.23 NS
Urea umol/L	Mean ± SE	38.20 ± 1.06	43.00 ± 0.70	37.00 ± 1.58	37.20 ± 1.49
	*t*-test		−4.49**	4.04**	4.39**

Significant differences with controls calculated by paired sample *t*-test. *means significant at *P* < 0.05, **means highly significant at *P* < 0.01, ***means very high significant at *P* < 0.001, and NS means nonsignificant.

**Table 4:** Effect of oat bran and barley bran supplementation for 8 weeks on antioxidant and oxidant enzymes in rats with induced hypercholesterolemia.

Parameters	Statistics	G1	G2	G3	G4
GST U/g.tissue	Mean ± SE	24.10 ± 0.26	22.20 ± 0.21	23.86 ± 1.23	25.38 ± 0.35
	*t*-test		12.52***	0.06 NS	−0.60 NS
Catalase U/g.tissue	Mean ± SE	164.44 ± 3.03	158.10 ± 2.42	173.32 ± 1.70	176.12 ± 5.71
	*t*-test		5.27***	−7.20***	−8.46 NS***
Lipid peroxide nmol/g.tissue	Mean ± SE	1057.40 ± 71.72	1058.30 ± 1.79	1001.80 ± 73.90	837.20 ± 60.98
	*t*-test		−0.01 NS	0.75 NS	3.62*

Significant differences with controls calculated by paired sample *t*-test. *means significant at *P* < 0.05, **means highly significant at *P* < 0.01, ***means very high significant at *P* < 0.001, and NS means nonsignificant.

## Abbreviations

CBC: Complete blood count
CHD: Coronary heart diseases
EDTA: Ethylenediaminetetraacetic acid
G: Glomerulus
G1: Fed normal diet
G2: Fed 1.0% w/w cholesterol in the diet
G3: Fed 1.0% w/w cholesterol and supplemented with 10% oat bran
G4: Fed 1.0% w/w cholesterol as in (G2) and supplemented with 10% barley bran
GST: Glutathione S-transferase
H&S: Hematoxylin and eosin
LDL: Low density lipoproteins
LDL-C: Low density lipoproteins cholesterol
s.LDL-C: Serum low density lipoproteins cholesterol
s.TC: Serum total cholesterol
s.TG: Serum triglyceride
s.VLDL-C: Serum very low density lipoprotein-cholesterol
SE: Standard errors
SPSS: Statistical Program for Sociology Scientists
TC: Total cholesterol.
